# *N*-(Pyrimidin-2-yl)aniline

**DOI:** 10.1107/S1600536809007685

**Published:** 2009-03-06

**Authors:** Edura Badaruddin, Nasir Shah Bakhtiar, Zaharah Aiyub, Zanariah Abdullah, Seik Weng Ng

**Affiliations:** a Department of Chemistry, University of Malaya, 50603 Kuala Lumpur, Malaysia

## Abstract

There are two molecules in the asymmetric unit of the title compound, C_10_H_9_N_3_, with inter-ring dihedral angles of 31.1 (1) and 35.3 (1)°. The bridging C—N—C bond angles are 128.2 (1) and 129.1 (1)°. In the crystal, the two independent mol­ecules are linked into a dimer by two N—H⋯N hydrogen bonds.

## Related literature

For the structure of 4-chloro-*N*-(pyrimidin-2-yl)aniline, see: Maizathul Akmam *et al.* (2009 ▶).
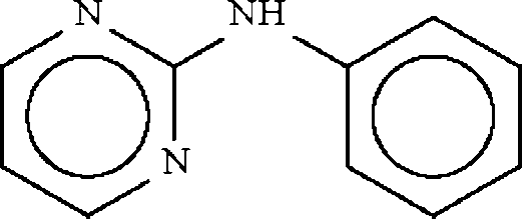


## Experimental

### 

#### Crystal data

C_10_H_9_N_3_
*M* *_r_* = 171.20
Triclinic, 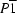

*a* = 8.8792 (2) Å
*b* = 9.9382 (2) Å
*c* = 10.2038 (2) Å
α = 93.186 (1)°
β = 103.665 (1)°
γ = 97.780 (1)°
*V* = 863.28 (3) Å^3^
*Z* = 4
Mo *K*α radiation
μ = 0.08 mm^−1^
*T* = 123 K
0.35 × 0.20 × 0.10 mm

#### Data collection

Bruker SMART APEX diffractometer
Absorption correction: none
8238 measured reflections
3950 independent reflections
3144 reflections with *I* > 2σ(*I*)
*R*_int_ = 0.020

#### Refinement

*R*[*F* ^2^ > 2σ(*F* ^2^)] = 0.039
*wR*(*F* ^2^) = 0.108
*S* = 1.03
3950 reflections
243 parameters
2 restraints
H atoms treated by a mixture of independent and constrained refinement
Δρ_max_ = 0.20 e Å^−3^
Δρ_min_ = −0.23 e Å^−3^

### 

Data collection: *APEX2* (Bruker, 2008 ▶); cell refinement: *SAINT* (Bruker, 2008 ▶); data reduction: *SAINT*; program(s) used to solve structure: *SHELXS97* (Sheldrick, 2008 ▶); program(s) used to refine structure: *SHELXL97* (Sheldrick, 2008 ▶); molecular graphics: *X-SEED* (Barbour, 2001 ▶); software used to prepare material for publication: *publCIF* (Westrip, 2009 ▶).

## Supplementary Material

Crystal structure: contains datablocks global, I. DOI: 10.1107/S1600536809007685/xu2492sup1.cif

Structure factors: contains datablocks I. DOI: 10.1107/S1600536809007685/xu2492Isup2.hkl

Additional supplementary materials:  crystallographic information; 3D view; checkCIF report

## Figures and Tables

**Table 1:** Hydrogen-bond geometry (Å, °)

*D*—H⋯*A*	*D*—H	H⋯*A*	*D*⋯*A*	*D*—H⋯*A*
N1—H1⋯N5	0.89 (1)	2.10 (1)	2.972 (1)	164 (1)
N4—H4⋯N2	0.89 (1)	2.15 (1)	3.020 (1)	165 (1)

**Fig. 1.**
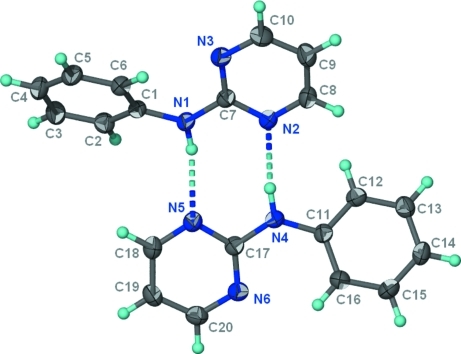
Thermal ellipsoid plot (Barbour, 2001) of the two independent molecules of C10H9N3 at the 70% probability level. Hydrogen atoms are drawn as spheres of arbitrary radius. Dashed lines denote hydrogen bonds.

## supplementary crystallographic information

### Experimental

2-Chloropyrimidine (0.05 mol), aniline (0.05 mol) and ethanol (5 ml) were heated at 423–433 K for 3 h. The product was dissolved in water and the solution extracted with ether. The ether phase was dried over sodium sulfate; the evaporation of the solvent gave well shaped colorless crystals along with some unidentified brown material.

### Refinement

Carbon-bound H-atoms were placed in calculated positions (C—H 0.95 Å) and were included in the refinement in the riding model approximation, with *U*_iso_(H) set to 1.2*U*_eq_(C). The amino H-atoms were located in a difference Fourier map, and were refined with a distance restraint of N–H 0.88±0.01 Å; their isotropic temperature factors were refined.

### Crystal data

**Table:**

C_10_H_9_N_3_	*Z* = 4
*M**_r_* = 171.20	*F*(000) = 360
Triclinic, *P*1	*D*_x_ = 1.317 Mg m^−^^3^
Hall symbol: -P 1	Mo *K*α radiation, λ = 0.71073 Å
*a* = 8.8792 (2) Å	Cell parameters from 3015 reflections
*b* = 9.9382 (2) Å	θ = 2.7–28.3°
*c* = 10.2038 (2) Å	µ = 0.08 mm^−^^1^
α = 93.186 (1)°	*T* = 123 K
β = 103.665 (1)°	Prism, colorless
γ = 97.780 (1)°	0.35 × 0.20 × 0.10 mm
*V* = 863.28 (3) Å^3^	

### Data collection

**Table:**

Bruker SMART APEX diffractometer	3144 reflections with *I* > 2σ(*I*)
Radiation source: fine-focus sealed tube	*R*_int_ = 0.020
graphite	θ_max_ = 27.5°, θ_min_ = 2.1°
ω scans	*h* = −11→11
8238 measured reflections	*k* = −12→12
3950 independent reflections	*l* = −13→12

### Refinement

**Table:**

Refinement on *F*^2^	Primary atom site location: structure-invariant direct methods
Least-squares matrix: full	Secondary atom site location: difference Fourier map
*R*[*F*^2^ > 2σ(*F*^2^)] = 0.039	Hydrogen site location: inferred from neighbouring sites
*wR*(*F*^2^) = 0.108	H atoms treated by a mixture of independent and constrained refinement
*S* = 1.03	*w* = 1/[σ^2^(*F*_o_^2^) + (0.056*P*)^2^ + 0.1316*P*] where *P* = (*F*_o_^2^ + 2*F*_c_^2^)/3
3950 reflections	(Δ/σ)_max_ = 0.001
243 parameters	Δρ_max_ = 0.20 e Å^−^^3^
2 restraints	Δρ_min_ = −0.22 e Å^−^^3^

### Fractional atomic coordinates and isotropic or equivalent isotropic displacement parameters (Å^2^)

**Table:**

	*x*	*y*	*z*	*U*_iso_*/*U*_eq_	
N1	0.63241 (11)	0.69760 (10)	0.39973 (10)	0.0229 (2)	
H1	0.5458 (13)	0.7328 (15)	0.4021 (15)	0.042 (4)*	
N2	0.60532 (11)	0.62876 (10)	0.60461 (10)	0.0235 (2)	
N3	0.82465 (11)	0.58169 (10)	0.51919 (10)	0.0257 (2)	
N4	0.33475 (12)	0.78540 (10)	0.59499 (10)	0.0256 (2)	
H4	0.4034 (15)	0.7306 (13)	0.5833 (14)	0.038 (4)*	
N5	0.37902 (11)	0.86480 (10)	0.39948 (10)	0.0256 (2)	
N6	0.18856 (11)	0.94997 (10)	0.50126 (10)	0.0245 (2)	
C1	0.69680 (13)	0.72230 (11)	0.28811 (11)	0.0212 (2)	
C2	0.59235 (14)	0.72964 (12)	0.16374 (12)	0.0259 (3)	
H2	0.4827	0.7163	0.1571	0.031*	
C3	0.64691 (15)	0.75616 (13)	0.04969 (12)	0.0305 (3)	
H3	0.5748	0.7621	−0.0342	0.037*	
C4	0.80703 (15)	0.77406 (13)	0.05803 (13)	0.0304 (3)	
H4A	0.8449	0.7896	−0.0203	0.036*	
C5	0.91044 (14)	0.76895 (12)	0.18192 (12)	0.0273 (3)	
H5	1.0200	0.7824	0.1882	0.033*	
C6	0.85757 (13)	0.74464 (11)	0.29708 (12)	0.0234 (2)	
H6	0.9304	0.7432	0.3816	0.028*	
C7	0.69174 (13)	0.63447 (11)	0.51132 (11)	0.0208 (2)	
C8	0.66215 (14)	0.56772 (12)	0.71431 (12)	0.0269 (3)	
H8	0.6055	0.5618	0.7825	0.032*	
C9	0.79969 (15)	0.51238 (14)	0.73406 (13)	0.0321 (3)	
H9	0.8393	0.4706	0.8140	0.039*	
C10	0.87631 (14)	0.52129 (13)	0.63096 (13)	0.0309 (3)	
H10	0.9700	0.4826	0.6402	0.037*	
C11	0.27870 (12)	0.77031 (12)	0.71236 (11)	0.0219 (2)	
C12	0.28250 (13)	0.64404 (12)	0.76560 (12)	0.0245 (3)	
H12	0.3171	0.5728	0.7199	0.029*	
C13	0.23654 (14)	0.62121 (13)	0.88390 (12)	0.0281 (3)	
H13	0.2399	0.5349	0.9191	0.034*	
C14	0.18554 (15)	0.72450 (14)	0.95093 (13)	0.0322 (3)	
H14	0.1532	0.7093	1.0319	0.039*	
C15	0.18214 (16)	0.84974 (14)	0.89877 (13)	0.0336 (3)	
H15	0.1470	0.9204	0.9447	0.040*	
C16	0.22895 (14)	0.87466 (12)	0.78070 (12)	0.0274 (3)	
H16	0.2271	0.9617	0.7469	0.033*	
C17	0.29837 (13)	0.87077 (11)	0.49672 (11)	0.0220 (2)	
C18	0.34955 (14)	0.95120 (12)	0.30445 (12)	0.0277 (3)	
H18	0.4052	0.9520	0.2356	0.033*	
C19	0.24158 (14)	1.03965 (12)	0.30177 (12)	0.0281 (3)	
H19	0.2233	1.1016	0.2342	0.034*	
C20	0.16165 (14)	1.03296 (12)	0.40278 (12)	0.0271 (3)	
H20	0.0840	1.0901	0.4020	0.033*	

### Atomic displacement parameters (Å^2^)

**Table:**

	*U*^11^	*U*^22^	*U*^33^	*U*^12^	*U*^13^	*U*^23^
N1	0.0200 (5)	0.0288 (5)	0.0229 (5)	0.0082 (4)	0.0084 (4)	0.0053 (4)
N2	0.0250 (5)	0.0245 (5)	0.0232 (5)	0.0057 (4)	0.0090 (4)	0.0032 (4)
N3	0.0237 (5)	0.0303 (5)	0.0251 (5)	0.0093 (4)	0.0068 (4)	0.0043 (4)
N4	0.0280 (5)	0.0276 (5)	0.0273 (5)	0.0120 (4)	0.0134 (4)	0.0068 (4)
N5	0.0250 (5)	0.0288 (5)	0.0260 (5)	0.0065 (4)	0.0102 (4)	0.0052 (4)
N6	0.0223 (5)	0.0262 (5)	0.0260 (5)	0.0066 (4)	0.0062 (4)	0.0031 (4)
C1	0.0246 (5)	0.0192 (5)	0.0215 (5)	0.0043 (4)	0.0086 (4)	0.0019 (4)
C2	0.0239 (6)	0.0279 (6)	0.0260 (6)	0.0052 (5)	0.0055 (5)	0.0043 (5)
C3	0.0364 (7)	0.0325 (7)	0.0221 (6)	0.0062 (5)	0.0054 (5)	0.0046 (5)
C4	0.0393 (7)	0.0294 (6)	0.0262 (6)	0.0035 (5)	0.0159 (5)	0.0041 (5)
C5	0.0273 (6)	0.0255 (6)	0.0319 (7)	0.0025 (5)	0.0140 (5)	0.0014 (5)
C6	0.0230 (5)	0.0241 (6)	0.0232 (6)	0.0032 (4)	0.0063 (4)	0.0007 (4)
C7	0.0208 (5)	0.0207 (5)	0.0206 (5)	0.0022 (4)	0.0055 (4)	−0.0003 (4)
C8	0.0319 (6)	0.0291 (6)	0.0220 (6)	0.0050 (5)	0.0107 (5)	0.0036 (5)
C9	0.0345 (7)	0.0394 (7)	0.0246 (6)	0.0114 (6)	0.0061 (5)	0.0114 (5)
C10	0.0263 (6)	0.0364 (7)	0.0317 (7)	0.0122 (5)	0.0055 (5)	0.0073 (5)
C11	0.0182 (5)	0.0257 (6)	0.0228 (6)	0.0043 (4)	0.0060 (4)	0.0035 (4)
C12	0.0245 (6)	0.0243 (6)	0.0259 (6)	0.0054 (4)	0.0080 (5)	0.0010 (5)
C13	0.0299 (6)	0.0269 (6)	0.0294 (6)	0.0042 (5)	0.0096 (5)	0.0075 (5)
C14	0.0363 (7)	0.0406 (7)	0.0259 (6)	0.0105 (6)	0.0160 (5)	0.0087 (5)
C15	0.0417 (7)	0.0368 (7)	0.0295 (7)	0.0172 (6)	0.0166 (6)	0.0036 (5)
C16	0.0328 (6)	0.0253 (6)	0.0275 (6)	0.0097 (5)	0.0104 (5)	0.0048 (5)
C17	0.0199 (5)	0.0228 (6)	0.0232 (6)	0.0025 (4)	0.0060 (4)	0.0017 (4)
C18	0.0291 (6)	0.0300 (6)	0.0254 (6)	0.0028 (5)	0.0102 (5)	0.0049 (5)
C19	0.0329 (6)	0.0254 (6)	0.0257 (6)	0.0047 (5)	0.0054 (5)	0.0074 (5)
C20	0.0268 (6)	0.0257 (6)	0.0287 (6)	0.0082 (5)	0.0042 (5)	0.0019 (5)

### Geometric parameters (Å, °)

**Table:**

N1—C7	1.3613 (14)	C5—H5	0.9500
N1—C1	1.4090 (14)	C6—H6	0.9500
N1—H1	0.892 (9)	C8—C9	1.3829 (17)
N2—C8	1.3294 (15)	C8—H8	0.9500
N2—C7	1.3555 (14)	C9—C10	1.3819 (17)
N3—C10	1.3322 (15)	C9—H9	0.9500
N3—C7	1.3423 (14)	C10—H10	0.9500
N4—C17	1.3584 (15)	C11—C16	1.3950 (16)
N4—C11	1.4093 (14)	C11—C12	1.3963 (16)
N4—H4	0.894 (8)	C12—C13	1.3831 (16)
N5—C18	1.3339 (15)	C12—H12	0.9500
N5—C17	1.3570 (14)	C13—C14	1.3859 (17)
N6—C20	1.3338 (15)	C13—H13	0.9500
N6—C17	1.3407 (14)	C14—C15	1.3818 (18)
C1—C6	1.3949 (15)	C14—H14	0.9500
C1—C2	1.3958 (16)	C15—C16	1.3883 (17)
C2—C3	1.3873 (16)	C15—H15	0.9500
C2—H2	0.9500	C16—H16	0.9500
C3—C4	1.3903 (18)	C18—C19	1.3824 (17)
C3—H3	0.9500	C18—H18	0.9500
C4—C5	1.3839 (18)	C19—C20	1.3824 (17)
C4—H4A	0.9500	C19—H19	0.9500
C5—C6	1.3860 (16)	C20—H20	0.9500
			
C7—N1—C1	128.19 (9)	C10—C9—H9	121.9
C7—N1—H1	114.6 (10)	C8—C9—H9	121.9
C1—N1—H1	117.0 (10)	N3—C10—C9	122.94 (11)
C8—N2—C7	115.61 (10)	N3—C10—H10	118.5
C10—N3—C7	116.06 (10)	C9—C10—H10	118.5
C17—N4—C11	129.13 (10)	C16—C11—C12	119.15 (10)
C17—N4—H4	115.5 (9)	C16—C11—N4	124.17 (10)
C11—N4—H4	115.4 (9)	C12—C11—N4	116.58 (10)
C18—N5—C17	115.71 (10)	C13—C12—C11	120.91 (11)
C20—N6—C17	115.97 (10)	C13—C12—H12	119.5
C6—C1—C2	119.10 (10)	C11—C12—H12	119.5
C6—C1—N1	123.56 (10)	C12—C13—C14	119.88 (11)
C2—C1—N1	117.30 (10)	C12—C13—H13	120.1
C3—C2—C1	120.70 (11)	C14—C13—H13	120.1
C3—C2—H2	119.7	C15—C14—C13	119.35 (11)
C1—C2—H2	119.7	C15—C14—H14	120.3
C2—C3—C4	120.04 (11)	C13—C14—H14	120.3
C2—C3—H3	120.0	C14—C15—C16	121.49 (11)
C4—C3—H3	120.0	C14—C15—H15	119.3
C5—C4—C3	119.13 (11)	C16—C15—H15	119.3
C5—C4—H4A	120.4	C15—C16—C11	119.20 (11)
C3—C4—H4A	120.4	C15—C16—H16	120.4
C4—C5—C6	121.39 (11)	C11—C16—H16	120.4
C4—C5—H5	119.3	N6—C17—N5	125.94 (11)
C6—C5—H5	119.3	N6—C17—N4	119.51 (10)
C5—C6—C1	119.59 (11)	N5—C17—N4	114.55 (10)
C5—C6—H6	120.2	N5—C18—C19	122.92 (11)
C1—C6—H6	120.2	N5—C18—H18	118.5
N3—C7—N2	125.92 (10)	C19—C18—H18	118.5
N3—C7—N1	119.10 (10)	C18—C19—C20	116.34 (11)
N2—C7—N1	114.96 (10)	C18—C19—H19	121.8
N2—C8—C9	123.22 (11)	C20—C19—H19	121.8
N2—C8—H8	118.4	N6—C20—C19	123.03 (11)
C9—C8—H8	118.4	N6—C20—H20	118.5
C10—C9—C8	116.22 (11)	C19—C20—H20	118.5
			
C7—N1—C1—C6	−31.08 (18)	C17—N4—C11—C16	−28.92 (18)
C7—N1—C1—C2	151.47 (11)	C17—N4—C11—C12	154.69 (11)
C6—C1—C2—C3	1.23 (17)	C16—C11—C12—C13	0.54 (17)
N1—C1—C2—C3	178.80 (10)	N4—C11—C12—C13	177.12 (10)
C1—C2—C3—C4	0.81 (18)	C11—C12—C13—C14	0.15 (18)
C2—C3—C4—C5	−1.85 (18)	C12—C13—C14—C15	−0.37 (19)
C3—C4—C5—C6	0.85 (18)	C13—C14—C15—C16	−0.1 (2)
C4—C5—C6—C1	1.19 (17)	C14—C15—C16—C11	0.8 (2)
C2—C1—C6—C5	−2.21 (17)	C12—C11—C16—C15	−1.00 (18)
N1—C1—C6—C5	−179.62 (10)	N4—C11—C16—C15	−177.30 (11)
C10—N3—C7—N2	−1.59 (17)	C20—N6—C17—N5	−2.37 (17)
C10—N3—C7—N1	−179.97 (10)	C20—N6—C17—N4	178.70 (10)
C8—N2—C7—N3	1.75 (16)	C18—N5—C17—N6	3.36 (17)
C8—N2—C7—N1	−179.81 (10)	C18—N5—C17—N4	−177.67 (10)
C1—N1—C7—N3	−3.22 (17)	C11—N4—C17—N6	−3.96 (18)
C1—N1—C7—N2	178.23 (10)	C11—N4—C17—N5	176.99 (11)
C7—N2—C8—C9	−0.28 (17)	C17—N5—C18—C19	−1.45 (17)
N2—C8—C9—C10	−1.14 (19)	N5—C18—C19—C20	−1.09 (18)
C7—N3—C10—C9	−0.06 (18)	C17—N6—C20—C19	−0.57 (17)
C8—C9—C10—N3	1.3 (2)	C18—C19—C20—N6	2.18 (18)

### Hydrogen-bond geometry (Å, °)

**Table:**

*D*—H···*A*	*D*—H	H···*A*	*D*···*A*	*D*—H···*A*
N1—H1···N5	0.89 (1)	2.10 (1)	2.972 (1)	164 (1)
N4—H4···N2	0.89 (1)	2.15 (1)	3.020 (1)	165 (1)

## References

[bb1] Barbour, L. J. (2001). *J. Supramol. Chem.* **1**, 189–191.

[bb2] Bruker (2008). *APEX2* and *SAINT* Bruker AXS Inc., Madison, Wisconsin, USA.

[bb3] Maizathul Akmam, A. B., Abdullah, Z. & Ng, S. W. (2009). *Acta Cryst.* E**65**, o94. 10.1107/S1600536808041184 PMC2968001 21581732

[bb4] Sheldrick, G. M. (2008). *Acta Cryst.* A**64**, 112–122. 10.1107/S0108767307043930 18156677

[bb5] Westrip, S. P. (2009). *publCIF* In preparation.

